# Supplementary material for: Myosin A and F-Actin play a critical role in mitochondrial dynamics and inheritance in Toxoplasma gondii
Source: bioRxiv. 2024 Mar 18:2024.03.18.585462. Preprint. [Version 1] doi: 10.1101/2024.03.18.585462 (PMC10983951; doi:10.1101/2024.03.18.585462)
Supplement: Supplement 1 [file NIHPP2024.03.18.585462v1-supplement-1.pdf]

bioRxiv preprint doi: https://doi.org/10.1101/2024.03.18.585462; this version posted March 18, 2024. The copyright holder for this preprint (which was not certified by peer review) is the author/funder, who has granted bioRxiv a license to display the preprint in perpetuity. It is made available under aCC-BY 4.0 International license.

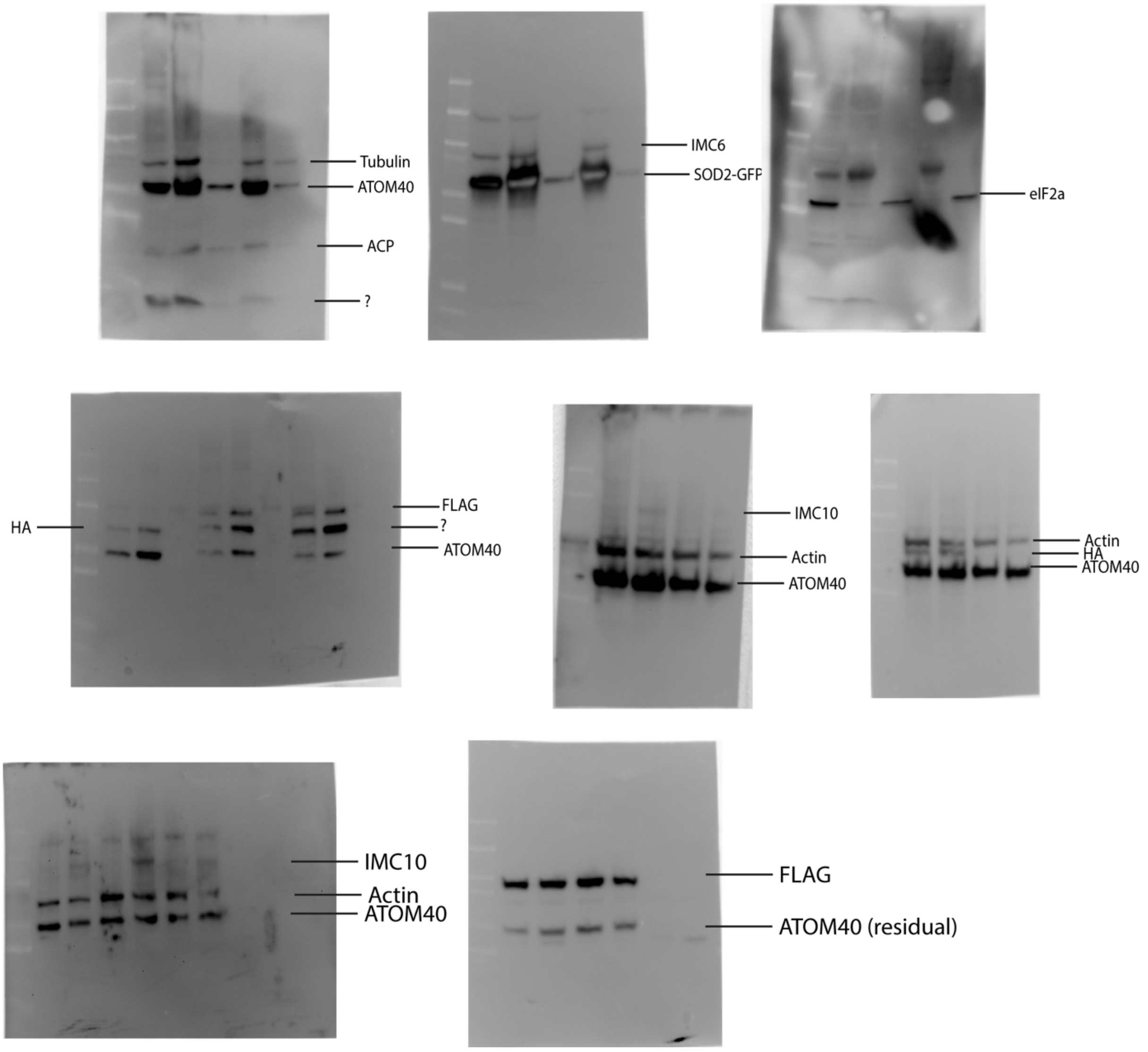

Figure S1 – Raw images of western blots. Western blots of Figure 6 (A) and Figure 10 (B).

bioRxiv preprint doi: https://doi.org/10.1101/2024.03.18.585462; this version posted March 18, 2024. The copyright holder for this preprint (which was not certified by peer review) is the author/funder, who has granted bioRxiv a license to display the preprint in perpetuity. It is made available under aCC-BY 4.0 International license.

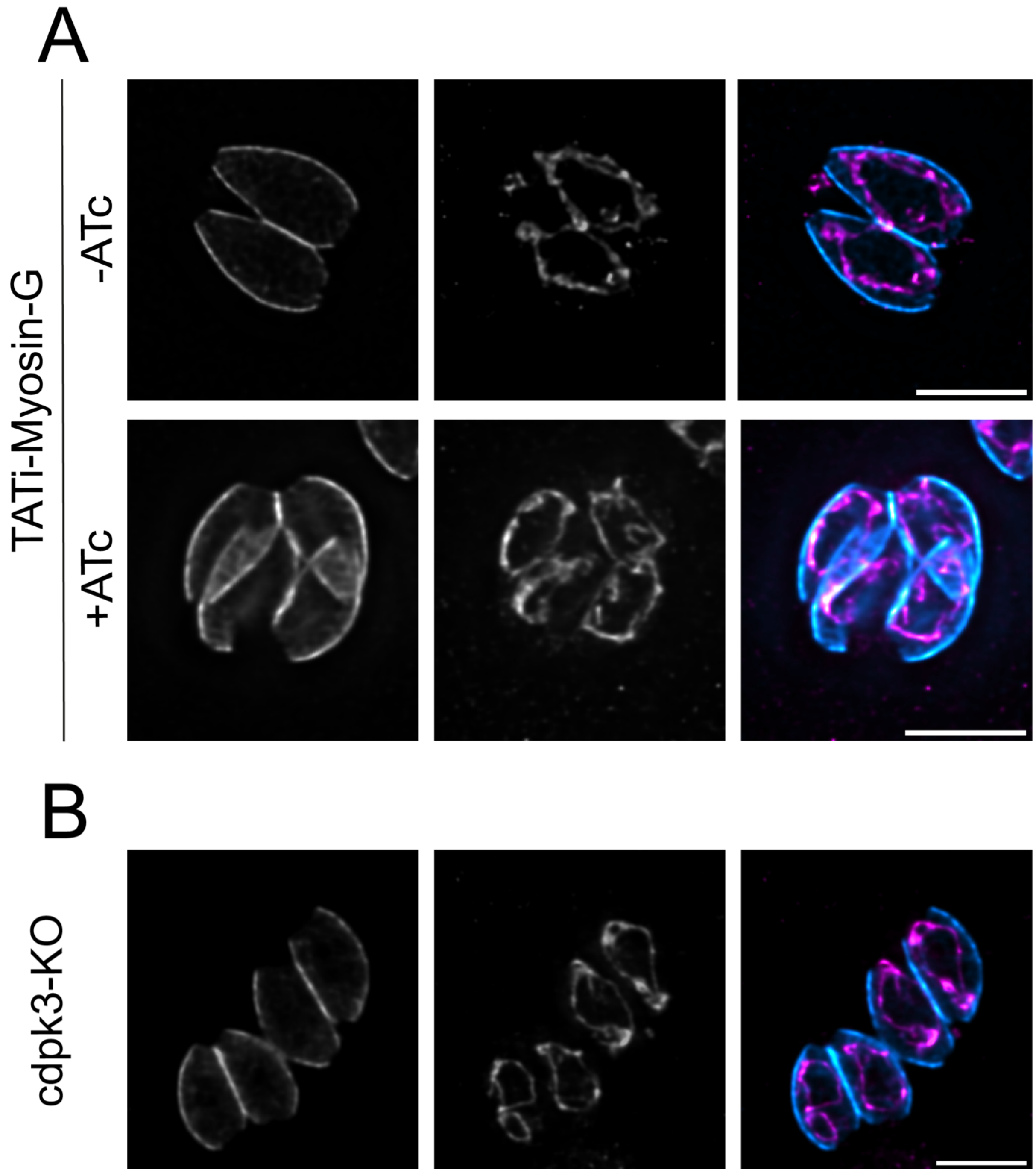

Figure S2 – Depletion of Myosin-G and CDPK3 does not affect mitochondrion morphology in intracellular parasites. Intracellular parasites of the TATi-Myosin-G (A) and cdpk3-KO (B) were grown in fibroblasts for 16 hours, then stained for IMC10 (cyan) and F_1B-ATPase, a mitochondrial marker (magenta). To induce the knockdown of Myosin-G, parasites were kept in the presence of anhydrotetracycline (ATc, 0.5 µg/ml) for 16h. Scale bar: 5 µm.

bioRxiv preprint doi: https://doi.org/10.1101/2024.03.18.585462; this version posted March 18, 2024. The copyright holder for this preprint (which was not certified by peer review) is the author/funder, who has granted bioRxiv a license to display the preprint in perpetuity. It is made available under aCC-BY 4.0 International license.

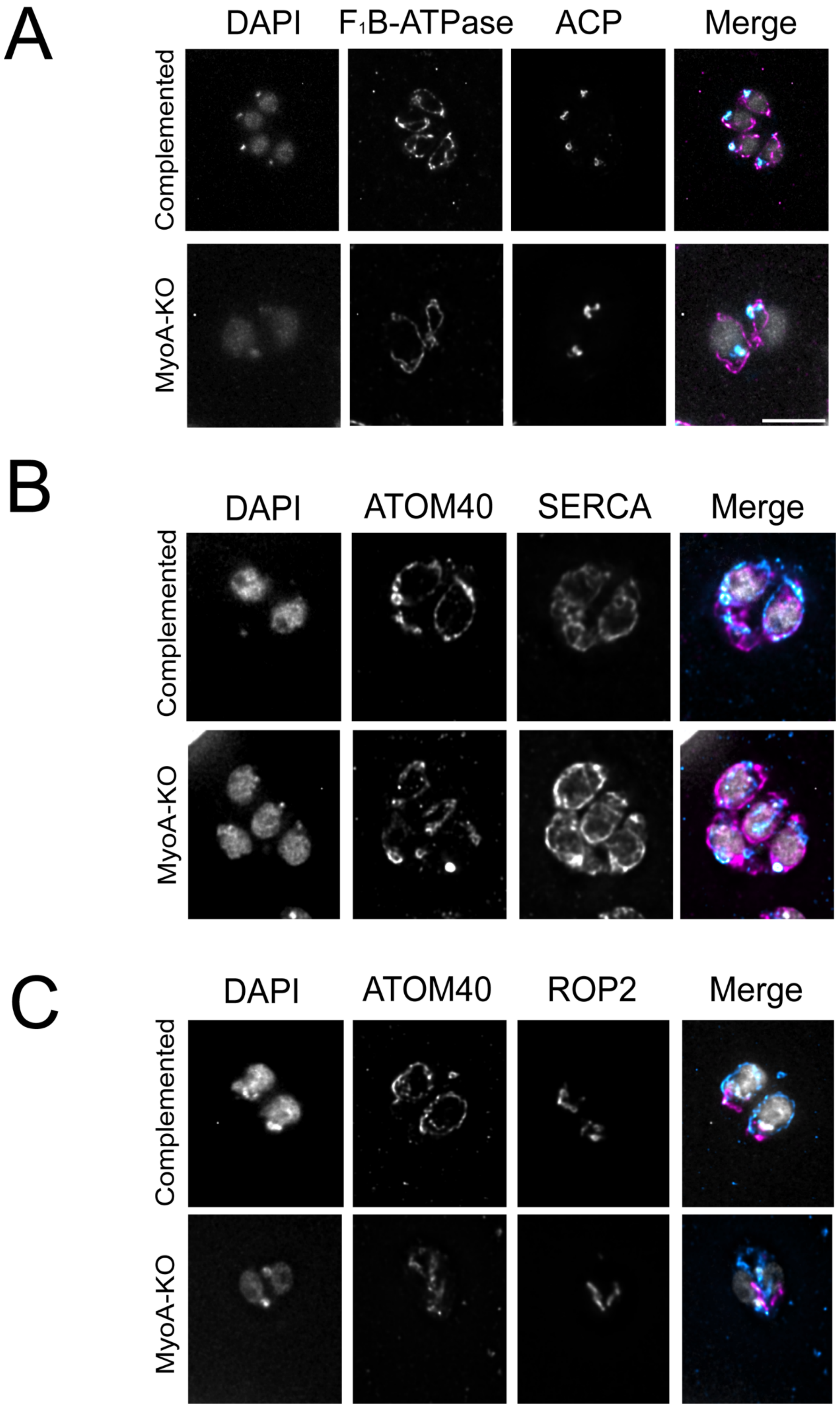

bioRxiv preprint doi: https://doi.org/10.1101/2024.03.18.585462; this version posted March 18, 2024. The copyright holder for this preprint (which was not certified by peer review) is the author/funder, who has granted bioRxiv a license to display the preprint in perpetuity. It is made available under aCC-BY 4.0 International license.

Figure S3. Depletion of Myosin-A does not affect apicoplast, endoplasmic reticulum, or rhoptries morphology in intracellular parasites. Intracellular parasites of the complement and myosin-A KO strains were grown in fibroblasts for 16 hours, then stained with anti-ACP to detect the apicoplast (A), anti-SERCA to detect the ER (B), or with anti-ROP2 to detect the rhoptriesROP2 (rhoptries). Either F_1B-ATPase (A) or ATOM40 (B and C) were used as a mitochondrial marker. Scale bar: 5 μm.

bioRxiv preprint doi: https://doi.org/10.1101/2024.03.18.585462; this version posted March 18, 2024. The copyright holder for this preprint (which was not certified by peer review) is the author/funder, who has granted bioRxiv a license to display the preprint in perpetuity. It is made available under aCC-BY 4.0 International license.

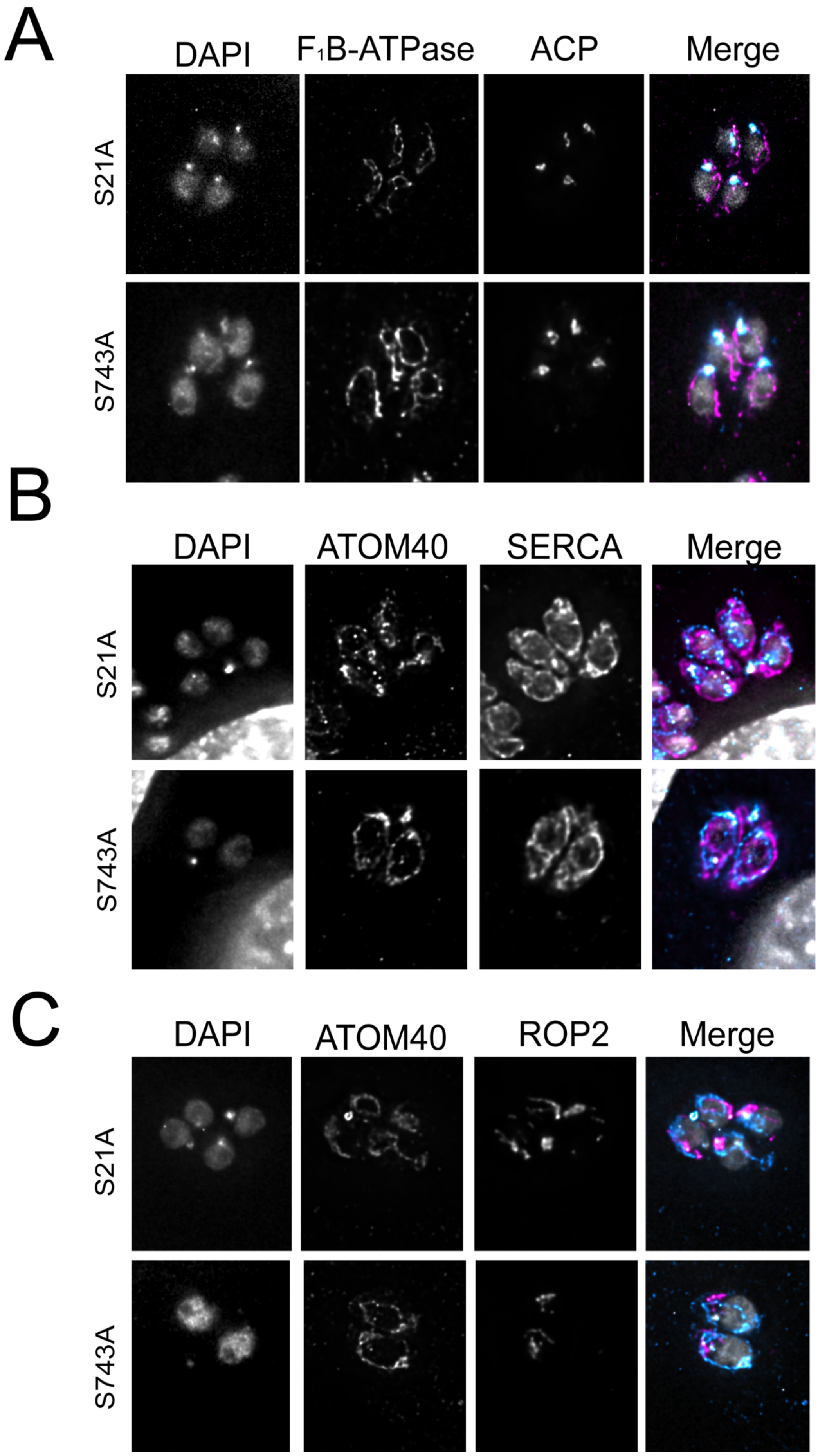

bioRxiv preprint doi: https://doi.org/10.1101/2024.03.18.585462; this version posted March 18, 2024. The copyright holder for this preprint (which was not certified by peer review) is the author/funder, who has granted bioRxiv a license to display the preprint in perpetuity. It is made available under aCC-BY 4.0 International license.

Figure S4. N- or C-terminal mutation of Myosin-A does not affect apicoplast, endoplasmic reticulum, or rhoptries morphology in intracellular parasites. Intracellular parasites expressing FLAG-Myosin-A S21A and FLAG-Myosin-A S743A were grown in fibroblasts for 16 hours.Cultures were then stained with anti-ACP to detect the apicoplast (A), anti-SERCA to detect the ER (B), or with anti-ROP2 to detect the rhoptriesROP2 (rhoptries). Either F_1B-ATPase (A) or ATOM40 (B and C) were used as a mitochondrial marker. Scale bar: 5 μm.

bioRxiv preprint doi: https://doi.org/10.1101/2024.03.18.585462; this version posted March 18, 2024. The copyright holder for this preprint (which was not certified by peer review) is the author/funder, who has granted bioRxiv a license to display the preprint in perpetuity. It is made available under aCC-BY 4.0 International license.

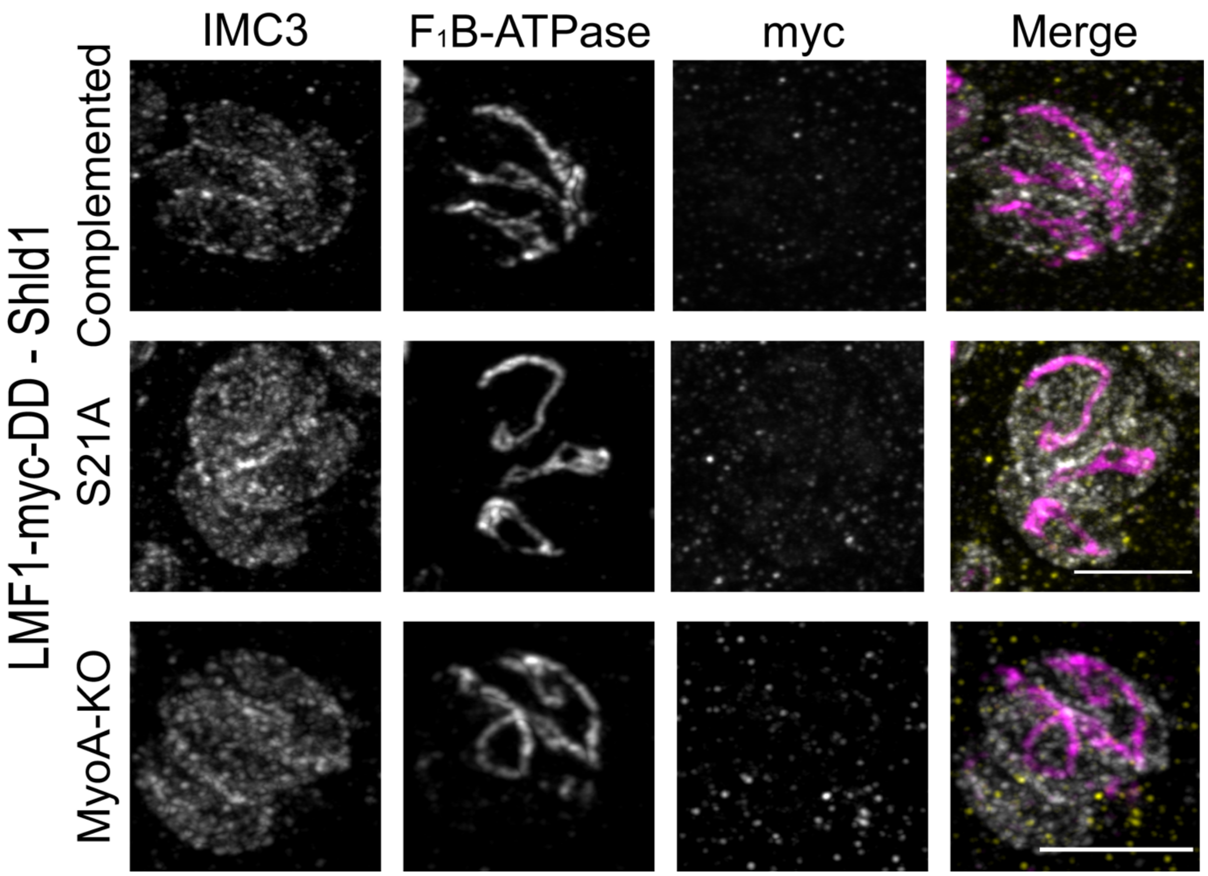

Figure S5. Establishment of parasite strains with conditional expression of LMF1. Representative IFA of MyoA complemented, MyoA S21A, or MyoA KO parasites expressing LMF1-myc-DD showing expression LMF1(Myc) in the absence of Shld1. Parasites were stained for IMC3 (gray), F_1B-ATPase (magenta), and myc (yellow). Scale bar: 5μm.

bioRxiv preprint doi: https://doi.org/10.1101/2024.03.18.585462; this version posted March 18, 2024. The copyright holder for this preprint (which was not certified by peer review) is the author/funder, who has granted bioRxiv a license to display the preprint in perpetuity. It is made available under aCC-BY 4.0 International license.

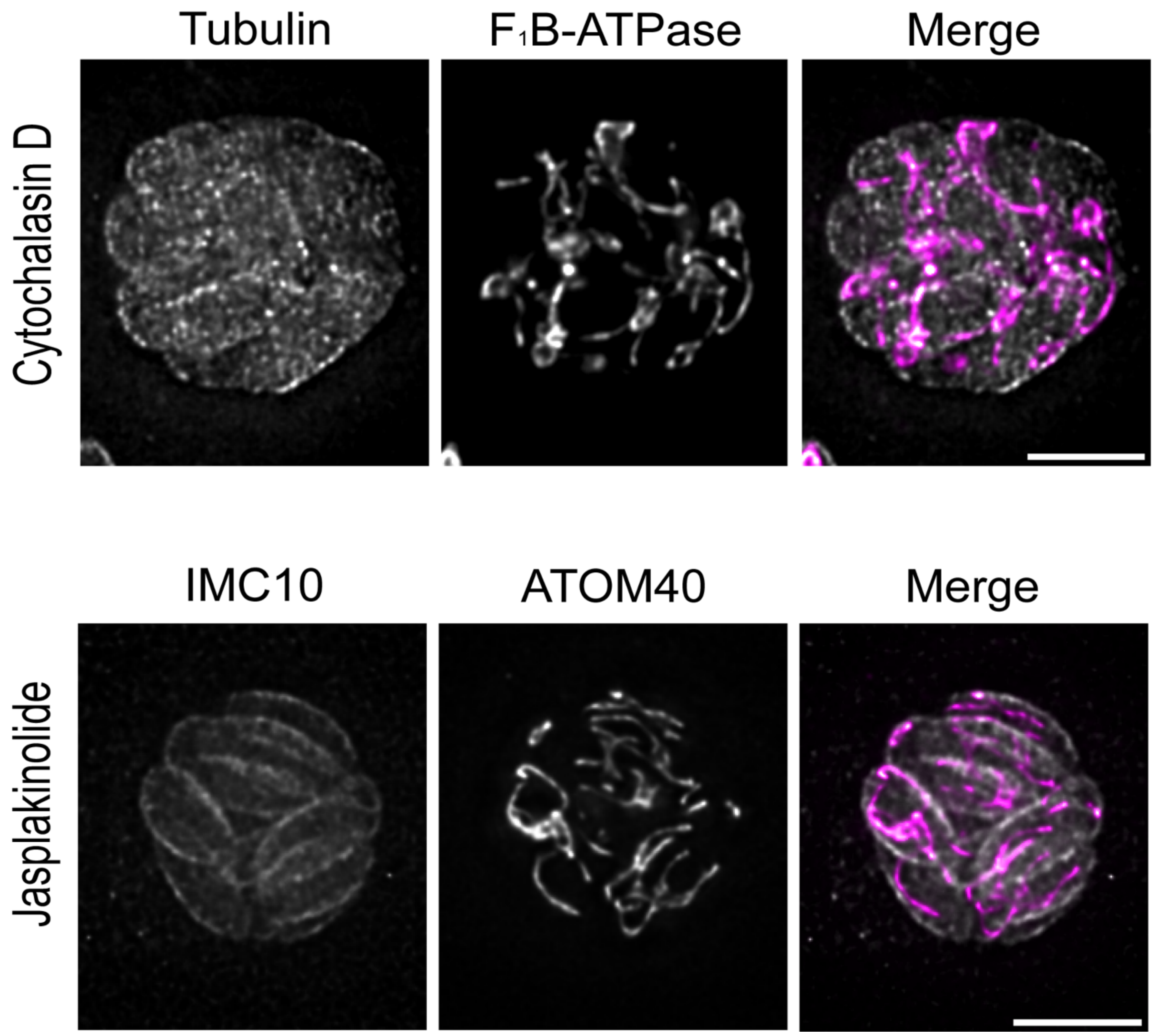

Figure S6. Actin is critical for mitochondrion morphology. IFA of large parasite vacuoles grown in the presence of 1 mM of Cytochalasin D (A) or 1 mM Jasplakinolide (B). Parasites were stained for Tubulin (gray) and F_1B-ATPase (magenta) or IMC10 (gray) and ATOM40 (magenta). Scale bar: 5μm.

bioRxiv preprint doi: https://doi.org/10.1101/2024.03.18.585462; this version posted March 18, 2024. The copyright holder for this preprint (which was not certified by peer review) is the author/funder, who has granted bioRxiv a license to display the preprint in perpetuity. It is made available under aCC-BY 4.0 International license.

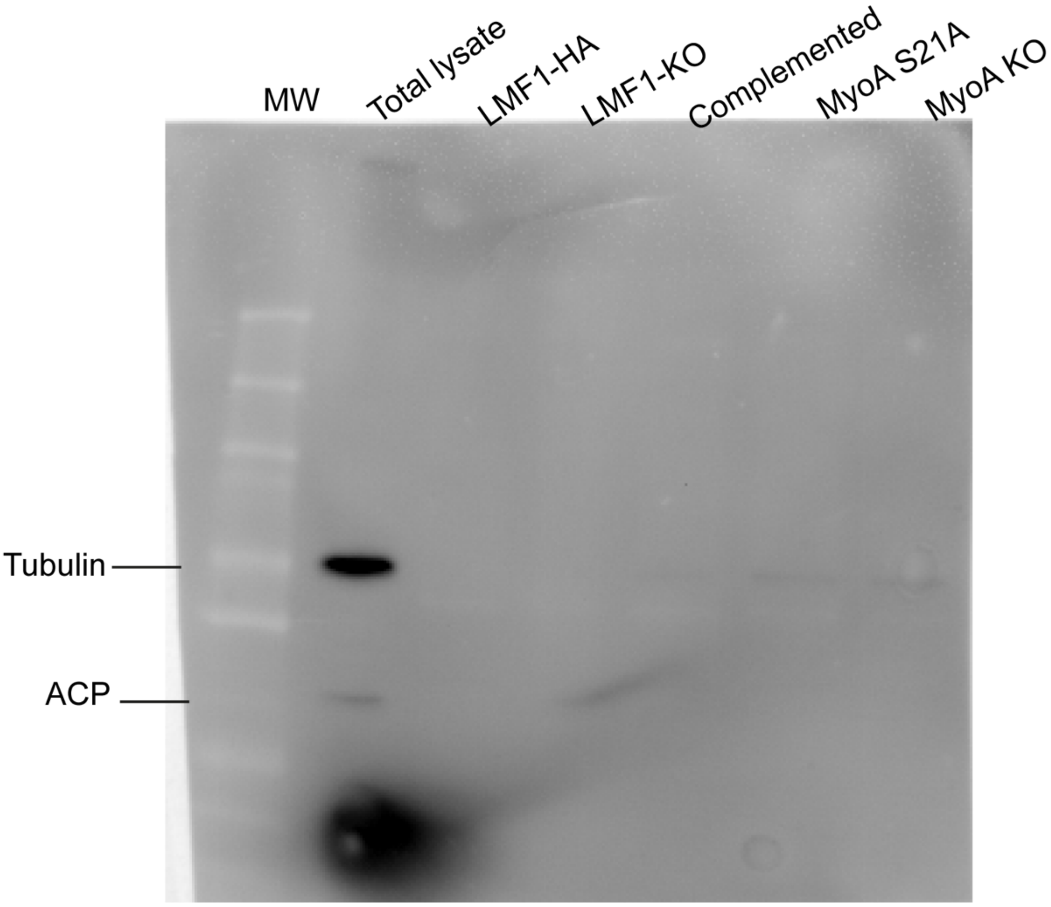

Figure S7. Enriched mitochondrion fractions do not carry microtubules and apicoplasts. Membranes of post-cushion enriched mitochondrion vesicles were probed for the presence of microtubules (anti-Tubulin) or apicoplast (anti-ACP).
